# *Schistosoma japonicum* infection associated with membranous nephropathy: a case report

**DOI:** 10.1186/s12879-022-07092-0

**Published:** 2022-02-05

**Authors:** Zhou-Ning Liao, Li-Jian Tao, Hong-ling Yin, Xiang-Chen Xiao, Min-Xiang Lei, Zhang-Zhe Peng

**Affiliations:** 1 Department of Nephrology, Xiangya Hospital, Central South University, Changsha, 410008 Hunan China; 2 Department of Nephropathology, Xiangya Hospital, Central South University, Changsha, China; 3 Hunan Key Lab of Organ Fibrosis, Changsha, China

**Keywords:** *Schistosoma japonicum*, Membranous nephropathy, Nephrotic syndrome

## Abstract

**Background:**

Schistosomiasis is one of the most contagious parasitic diseases affecting humans; however, glomerular injury is a rare complication mainly described with *Schistosoma mansoni* infection. We report a case of membranous nephropathy associated with *Schistosoma japonicum* infection in a Chinese man.

**Case presentation:**

A 51-year-old Chinese male with a long history of *S. japonicum* infection presented to the hospital with a slowly progressing severe lower limb edema and foaming urine for over 5 months. Serum *S. japonicum*antigen test was positive and immunohistochemistry showed that the glomeruli were positive for the antigens. The renal pathologic diagnosis was stage III membranous nephropathy. The patient was treated with glucocorticoid, praziquantel, and an angiotensin-converting enzyme inhibitor. The edema in both lower limbs disappeared within 2 weeks, but his renal function declined progressively and proteinuria persisted after 5 months of therapy.

**Conclusions:**

Different classes of schistosomal glomerulopathy have completely different clinical manifestation and prognosis. Therefore, efforts should focus on alleviating symptoms, prevention, and early detection. *S. japonicum*associated with membranous nephropathy may show a good curative effect and prognosis. However, it is necessary to monitor the renal function in such patients.

## Background

Schistosomiasis is one of the most infectious parasite illnesses that affects humans, killing 20,000 people per year [1, 2]. Glomerular damage is a very uncommon consequence associated with Schistosoma infections. The southern and eastern parts of China are endemic for schistosomiasis, while renal consequences are unusual. In China, schistosomiasis is endemic to the southern and eastern regions, but renal complications have rarely been reported. Three major species of *Schistosoma* affect ~ 200 million people: *Schistosoma hematobium* in Africa, *Schistosoma mansoni* in Africa and South America, and *Schistosoma japonicum* in the Far East [1]. Glomerular diseases had been well described in association with *S. mansoni* but exceptionally with other species [3]. Renal impairment is a severe form of schistosomiasis and the most common clinical presentation is nephrotic syndrome. According to the African Association of Nephrology (AFRAN), five classes of schistosomal glomerulopathy are recognized [4], and the most common histological finding is membranoproliferative glomerulonephritis (MPGN). The second most frequent histological type is focal segmental glomerulosclerosis [5]. The five classes do not include membranous nephropathy and only one case of *S. mansoni,* which caused membranous nephropathy, has been reported to date [6]. We report the first case of *S. japonicum* associated with membranous nephropathy in a Chinese man. We received written consent from the patient to publish the age, image findings, and pathologic pictures associated with this case report.

## Case presentation

**Fig. 1:**
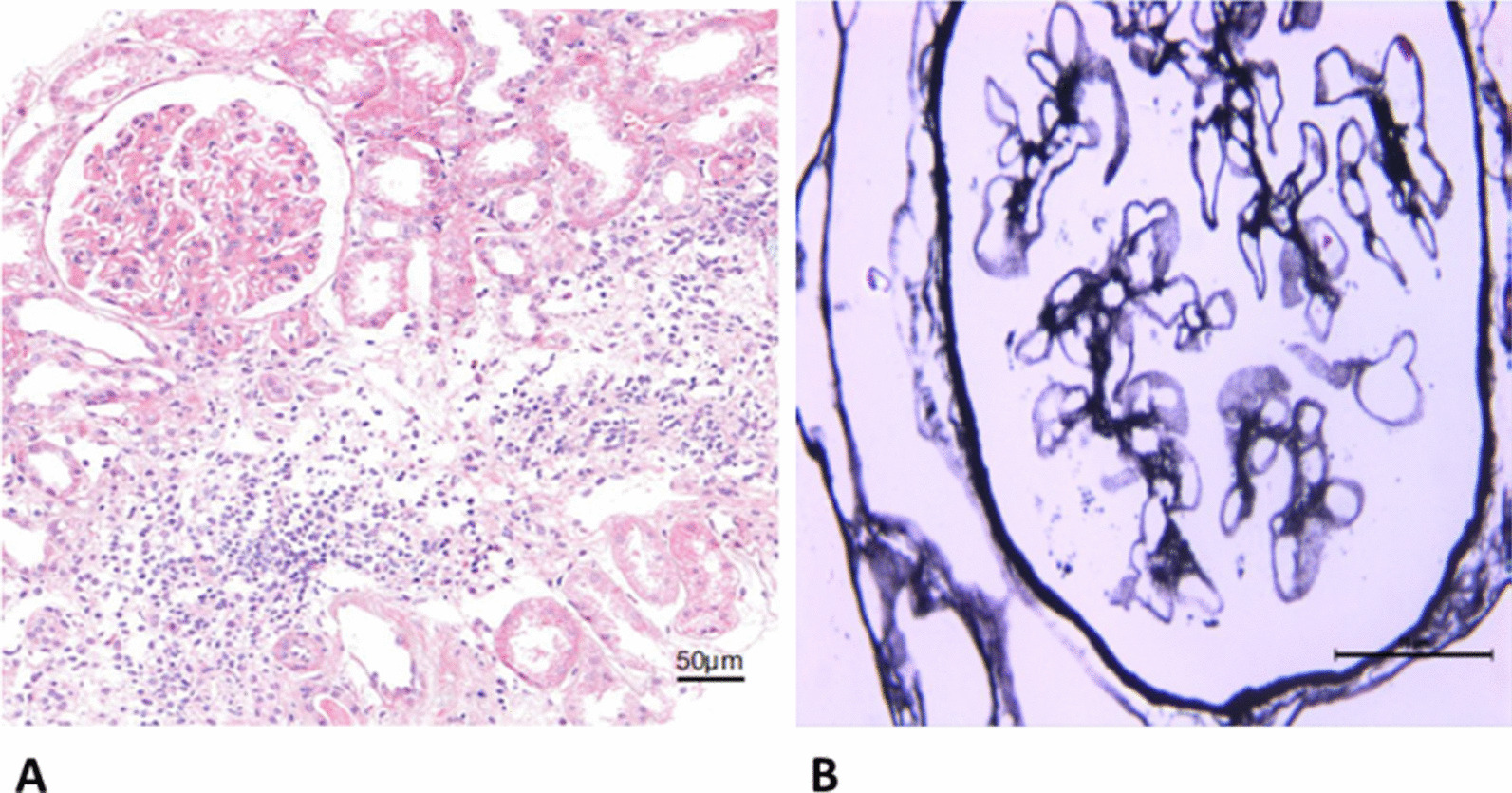
**A** Light microscopy: diffuse moderate to severe mesangial hypercellularity, interstitial infiltration of lymphocytes and fibrosis (H&E, × 200). **B** Extensive thickening with some spikes of the basement membrane (PASM, × 400)

**Fig. 2:**
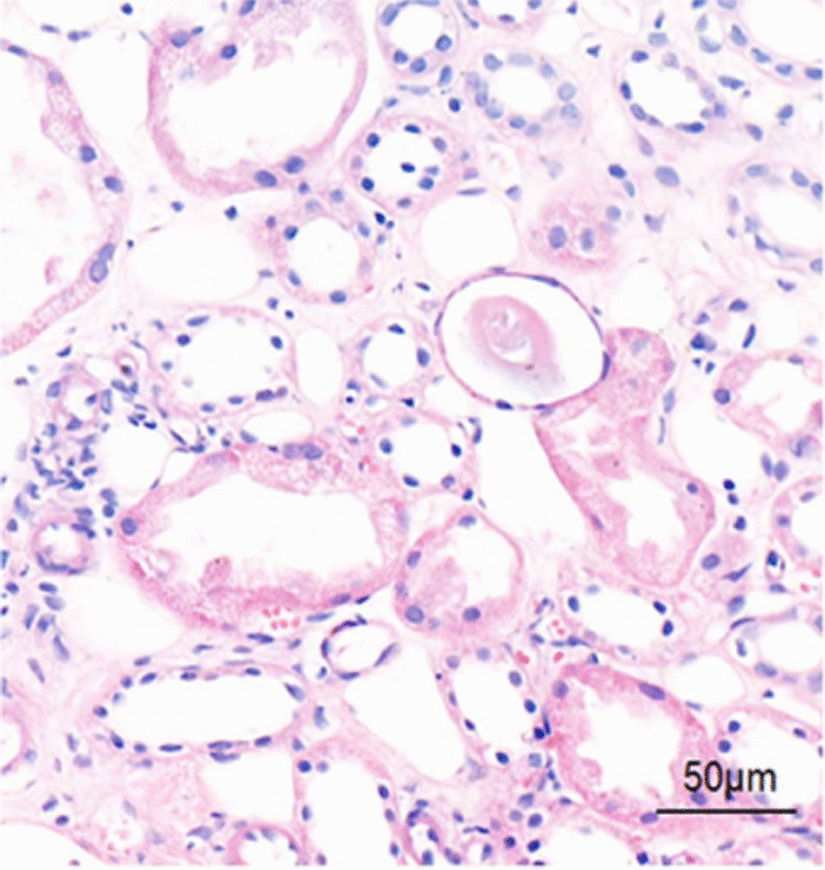
A foreign body around an elliptical structure in a renal proximal tubule was seen, consistent with a suspended *S. japonicum* egg (H&E, × 200)

**Fig. 3:**
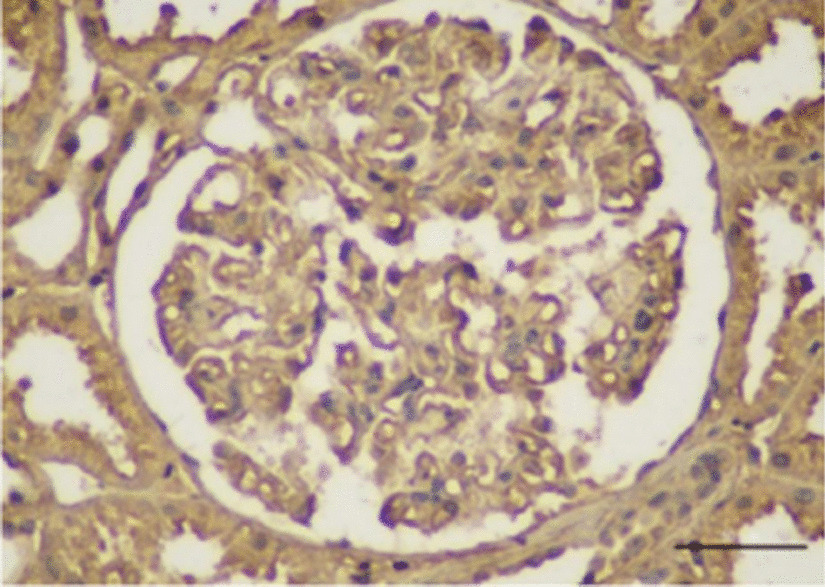
Immunohistochemical finding. *S. japonicum* antigen in the renal glomeruli and tubules was positive. Recognized by polyclonal antibodies IgG in sera of mice at 60 day post-infected (× 400)

**Fig. 4:**
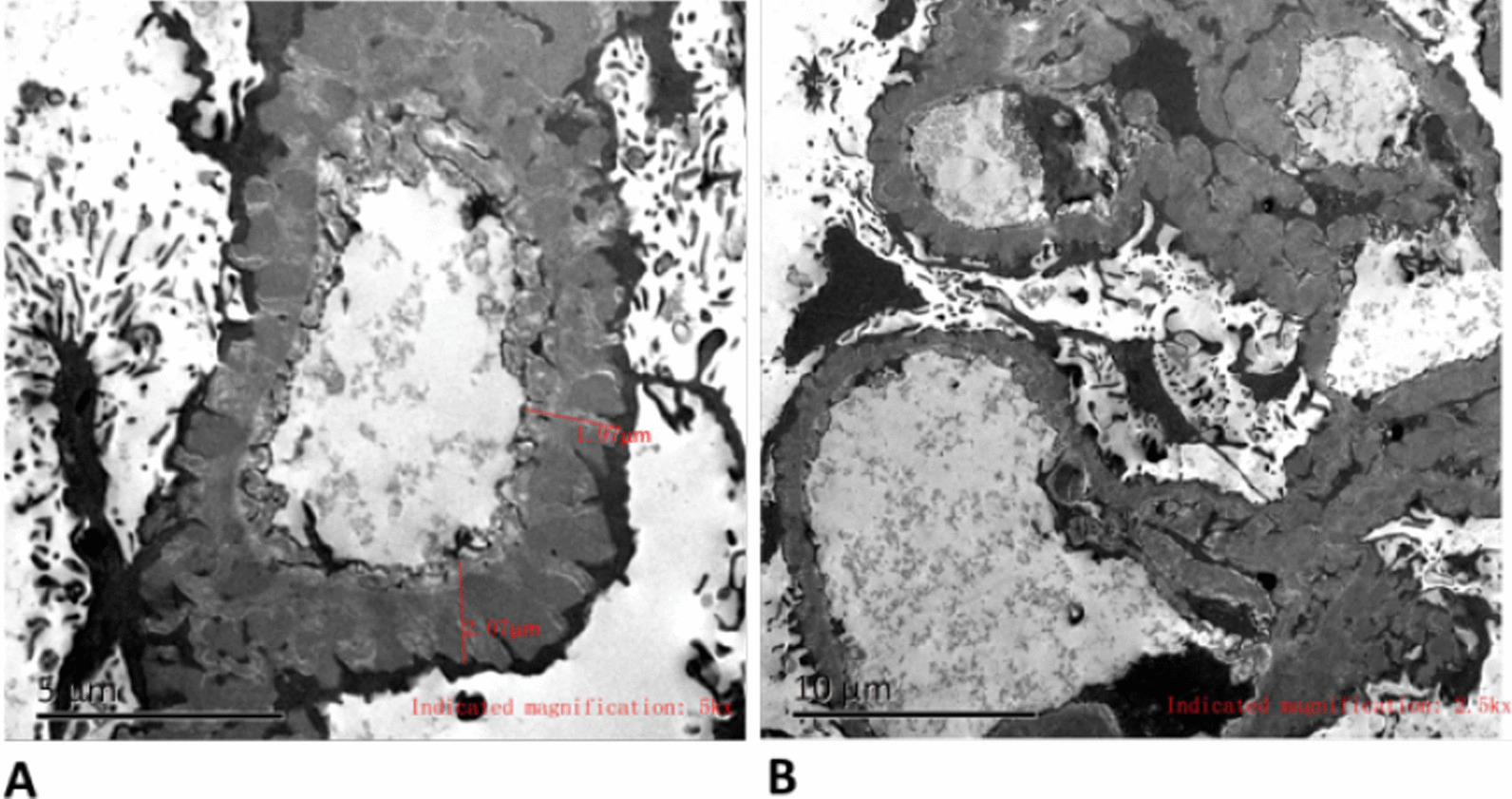
Electron microscopy. **A** Glomerulus had mild mesangial hyperplasia and thickened basement membrane (× 5000). **B** Electron density was deposited in the subepithelium and basement membrane of the glomerulus (× 2500)

A 51-year-old Chinese male presenting with slowly progressing severe lower limb edema and foaming urine for over 5 months was admitted to Xiangya Hospital. The patient was diagnosed with *S. japonicum* infection in 2000 due to Schistosomiasis eggs found in his feces. The patient had no personal or family history of renal disease. Physical examination at current hospital admission revealed severe edema of the lower extremities. Laboratory tests revealed creatinine, 92 µmol/l; glomerular filtration rate, 98.59 ml/min; white blood cell count, 18.13 × 10^9^/l; eosinophil count, 10.09 × 10^9^/l; albumin, 27.50 g/l; triglycerides, 1.65 mmol/l; total cholesterol, 9.98 mmol/l; 24 h proteinuria, 2388.44 mg/24 h. Autoantibody detection and serologic test results were negative for hepatitis B, hepatitis C, and HIV, but positive for *Schistosoma* antibody. Anti-nuclear antibodies include anti-Sm, anti-Ro, anti-La, anti-RNP, and anti-dsDNA antibodies were negative. Serum complement levels were normal. Abdominal B type ultrasonography revealed splenomegaly, diffuse lesion in the liver parenchyma; left kidney, 103 × 54 mm; and right kidney, 97 × 43 mm. Anti-phospholipase A2 receptor antibodies were detected at 48.13 RU/ml. Light microscopy examination for renal biopsy sample revealed diffuse moderate to severe mesangial hypercellularity, extensive thickening with some spikes of the basement membrane, interstitial infiltration of lymphocytes, and fibrosis (Fig. 1). A foreign body around an elliptical structure in the renal proximal tubule was noted, consistent with the findings of a suspended *S. japonicum* egg (Fig. 2). Immunohistochemically, the glomeruli were positive for *S. japonicum* antigens (tested by polyclonal antibodies derived from the sera of mice infected with *S. japonicum*) (Fig. 3). Immunofluorescence showed granular deposits of lgG, lgM, lgA, C3, C1q, kappa, and lambda in the glomerular capillary loop. The final pathologic diagnosis was stage III membranous nephropathy (Fig. 4). The treatment of the patient was started with oral glucocorticoid (prednisone, 25 mg/day), praziquantel (60 mg/kg), an angiotensin-converting enzyme inhibitor (ACEI, 0.15 g/day) and ZhiLing capsula (0.75 g, thrice daily) with supportive therapy (salt-free diet, Etamsylate, vitamin K1). After 7 days of treatment, the patient was discharged from hospital. The patient was put on continuous oral ACEI (0.15 g/day) and Compound α-Ketoacid Tablets (2.52 g, thrice daily) at home for 5 months, the edema in both lower limbs disappeared within 2 weeks, but the renal function declined progressively and proteinuria persisted after 5 months of therapy (Table 1).

**Table 1:** Progression of the tests between March and July 2018

Tests/dates	Reference values	03.05.18	03.21.18	05.31.18	06.08.18	07.03.18	07.30.18
White blood cells (× 10^9^/l)	3.5–9.5	18.13	13.51	10.7	8.9	12.5	13.5
Platelet count (fL)	123–350	256	247	233	208	169	215
Eosinophils count (× 10^9^/l)	0.02-0.52	10.09	1.72	1	0.8	1.1	0.1
Eosinophils (%)	0.4–8.0	55.7	12.8	9.7	8.5	9	0.1
Hemoglobin vale (g/l)	130–175	149	129	129	119	119	110
Urea (mg/dl)	3.10–8.00	4.4	6.67	4.89	6.38	8.78	14.71
Creatinine (µmol/l)	41–111	92	98.3	114	105.4	121	140.8
Alumin (g/l)	40–55	27.5	27.77	36.5	35.7	37.3	37.7
Triglycerides (mmol/l)	< 1.70	1.65	6.67	1.35	1.31		
Total cholesterol (mmol/l)	< 5.18	9.98	9.97	6.97	6.86		

Search for abnormal elements/sediment in the urine						
Proteins		Positive (+++)	Positive (++++)	Positive (++)	Positive (++++)	Positive (+)
Red blood cells	0–5	55	7	7	19	2
Urine volume/24 h l	L	1.8		1.5		
Microalbuminuria (mg/24 h)	0–30	3599		1332		
24 h proteinuria (mg/24 h)	< 150	3588.4		1710.62		

## Discussion and conclusion

This is the first case report of membranous nephropathy associated with *S. japonicum* infection in a Chinese man.

Schistosoma infection may be underdiagnosed in endemic areas with high schistosomiasis prevalence due to a lack of comprehensive screening. Furthermore, in 10–15% of patients with *S. mansoni* infection, glomerular damage related to schistosomiasis has been documented, with MPGN being the most frequent histological type [7–9]. Direct deposition of schistosomal antigens causes glomerular damage in most cases. Moreover, immune complex (IC) deposition is the main mechanism underlying the different forms of schistosomal glomerulonephritis [10], which is associated with IC deposition in the sub-endothelial, sub-epithelial, and mesangial regions of the glomerulus, together with lgA aggregates and parasite antigens, as shown in the glomeruli of both experimental animals and people with membranoproliferative and mesangioproliferative glomerulonephritis [11]. Schistosomal glomerulopathy is a distinct disease entity, which has been identified with experimental [12], epidemiologic [13], post-mortem [14], and clinical evidence [4]. The AFRAN endorsed a clinicopathologic classification for schistosomal glomerulopathy in 1992, wherein 5 classes of schistosomal glomerulopathy were recognized. Class I is mesangial proliferative glomerulonephritis. Class II is an exudative glomerulonephritis; patients with this form are concomitantly infected by *Salmonella* and *Schistosoma* [4]. Class III is MPGN usually reported in Caucasian individuals, and Class IV is a focal proliferative/sclerosing lesion typically seen in African-origin individuals. Class V is renal amyloidosis of the AA type. Recently, The inclusion of Class VI to the AFRAN classification of schistosomal glomerulopathy has been recommended. Class VI is cryoglobulinemic glomerulonephritis associated with the hepatitis C virus infection.

Approximately, 10–15% of patients with the hepatosplenic form of the disease have renal involvement. Individuals with hepatosplenic schistosomiasis have greater laboratory and clinical indicators of renal impairment than patients with other clinical forms of *S. mansoni* infection or non-infected controls [15]. Although the present case of membranous nephropathy caused by *S. japonicum* infection was rare, the mechanism of glomerular damage may have been comparable to that caused by *S. mansoni* infection. Between 2003 and 2009, the Renal Pathology Services at the Goncalo Moniz Research Centre-Fiocruz conducted a study that indicated a decrease in the number of reports of *S. mansoni* infection in biopsy specimens. Positive results for *S. mansoni* was reported for 24 out of 689 patients, and 4 out of the 24 had membranous glomerulonephritis. The prevalence of schistosomal glomerulopathy has decreased as a result of widespread treatment with oral medicines [16]. Compared with the study between 2003 and 2006, a study reported by Queiroz between 1970 and 1973 revealed that positive results for *S. mansoni* infection were reported for 38 out of 100 individuals, and 2 of these 38 had membranous glomerulonephritis [11]. So far, one case of membranous nephropathy associated with *S. mansoni* infection has been reported by Neves et al. [6]; the renal biopsy results in this case supported the diagnosis of an organic renal lesion caused by *S. mansoni* infection.

Altogether*, S. japonicum* infection leading to membranous nephropathy appears to be unique and previously unreported. While anthelminthic and immunosuppressive drugs can relieve renal injury caused by schistosomiasis, the disease finally evolves to chronic end-stage renal disease (ESRD) [17]. Medical history, physical examination, laboratory examination, and renal pathology results in this report were consistent with a diagnosis of *S. japonicum* associated with membranous nephropathy. In this case, *S. japonicum* infection was induced by the patient's contact with infected water, as evidenced by the presence of Schistosomiasis eggs in the feces. Immunohistochemistry showed that the glomeruli were positive for parasitic antigens; treatment using praziquantel was effective. Moreover, since membranous nephropathy caused by other secondary factors was excluded, it can be inferred that the kidney injury was secondary to *S. japonicum* infection. Renal manifestations of glomerular disease caused by *S. mansoni* infection can range from asymptomatic albuminuria and normal renal function to chronic ESRD [7], although most patients have nephrotic syndrome and a plasma creatinine concentration between 88 and 176 µmol/l [8]. In individuals with class I–II disease, complete recovery can occur spontaneously or after therapy, while in cases with classes III–V, treatment with anthelmintic drugs and immunosuppressive agents is usually not effective to arrest the progression to ESRD. However, our patient’s case differed from all the above classes. During the 10-month follow-up, his plasma creatinine level progressively increased between March 2018 and July 2018 and thereafter remained stagnant between 110 and 120 µmol/l between July 2018 and December 2018; the proteinuria was persistent and showed no decline.

In summary, membranous nephropathy is a rare complication associated with *S. japonicum* infection. Following treatment with praziquantel, ACEI, and glucocorticoid, our patient's symptoms were rapidly resolved. However, the renal function declined progressively, creatinine and urea nitrogen levels increased, and proteinuria persisted. Therefore, efforts should be focused on alleviating symptoms, prevention, early detection, and treatment of *Schistosoma* infection among at-risk groups rather than eliminating urinary protein. Moreover, it is necessary to monitor the renal function in such cases.

Our research has several limitations, firstly, no attempt was made to give the patient the immunosuppressive therapy recommended for membranous nephropathy, such as rituximab, tacrolimus (FK506), because the patient did not have regular follow-up appointments. Secondly, it is not known whether the patient has progressed to end-stage renal disease because the follow-up period is not long enough.

## Data Availability

The datasets generated and analyzed during the current study are available from the corresponding author on reasonable request.

## Abbreviations

IC: Immune complexes
MPGN: Membranoproliferative glomerulonephritis
ESRD: End-stage renal disease
ACEI: Angiotensin converting enzyme inhibitors
AFRAN: African Association of Nephrology

## References

[1] Barsoum RS (2003). Schistosomiasis and the kidney. Semin Nephrol.

[2] Goncalves FO, Fontes TM, Canuto AP (2017). *Schistosoma mansoni* associated glomerulopathy with IgA mesangial deposits: case report. J Bras Nefrol.

[3] Gryseels B, Polman K, Clerinx J, Kestens L (2006). Human schistosomiasis. Lancet.

[4] Barsoum RS (1993). Schistosomal glomerulopathies. Kidney Int.

[5] Brito TD, Nussenzveig I, Carneiro CR, Silva AM (1999). *Schistosoma mansoni* associated glomerulopathy. Rev Inst Med Trop Sao Paulo.

[6] Neves PD, Bezerra KS, Silveira MA (2016). *Schistosoma mansoni* and membranous nephropathy. Kidney Int.

[7] Barsoum R (2004). The changing face of schistosomal glomerulopathy. Kidney Int.

[8] Martinelli R, Noblat AC, Brito E, Rocha H (1989). *Schistosoma mansoni*-induced mesangiocapillary glomerulonephritis: influence of therapy. Kidney Int.

[9] Nussenzveig I, De Brito T, Carneiro CR, Silva AM (2002). Human *Schistosoma mansoni*-associated glomerulopathy in Brazil. Nephrol Dial Transplant.

[10] Abensur H, Nussenzveig I, Saldanha LB (1992). Nephrotic syndrome associated with hepatointestinal schistosomiasis. Rev Inst Med Trop Sao Paulo.

[11] Dos-Santos WL, Sweet GM, Bahiense-Oliveira M, Rocha PN (2011). Schistosomal glomerulopathy and changes in the distribution of histological patterns of glomerular diseases in Bahia, Brazil. Mem Inst Oswaldo Cruz.

[12] Houba V (1979). Experimental renal disease due to schistosomiasis. Kidney Int.

[13] Ezzat E, Osman RA, Ahmet KY, Soothill JF (1974). The association between *Schistosoma haematobium* infection and heavy proteinuria. Trans R Soc Trop Med Hyg.

[14] Andrade ZA, Rocha H (1979). Schistosomal glomerulopathy. Kidney Int.

[15] Duarte DB, Vanderlei LA, Bispo RK (2014). Renal function in hepatosplenic schistosomiasis—an assessment of renal tubular disorders. PLoS ONE.

[16] Correia EI, Martinelli RP, Rocha H (1997). Is glomerulopathy due to *Schistosomiasis mansoni* disappearing?. Rev Soc Bras Med Trop.

[17] Seck SM, Sarr ML, Dial MC, Ka EF (2011). Schistosoma hematobium-associated glomerulopathy. Indian J Nephrol.

